# Incidence and treatment for hypomineralization of incisor and molar among school going Indian children

**DOI:** 10.6026/973206300200575

**Published:** 2024-05-31

**Authors:** Sajid khan, Shweta Sharma, Arunendra Singh Chauhan, Aiyana Parthi, Saima Ali, Mohd Amjad Tahseen

**Affiliations:** 1 Department of Pedodontics and Preventive Dentistry,Career Dental College, Lucknow, Uttar Pradesh, India; 2 Conservative Dentistry and Endodontics, Inderprastha Dental college, Ghaziabad, Uttar Pradesh, India; 3 Department of Public Health Dentistry, Maharishi Markandeshwar College of Dental Sciences and Research, Mullana, Haryana, India; 4 Department of Conservative Dentistry and Endodontics, Sri Sukhmani Dental College and Hospital, Derabassi, Punjab, India; 5 Department of Public Health Dentistry, Maharishi Markandeshwar College of Dental Sciences and Research, Mullana, Haryana, India; 6 Department Of Periodontics, Mithila Minority Dental College, Darbhanga, Bihar, India

**Keywords:** Molar incisor hypomineralization, children, school going children

## Abstract

The presence of molar incisor hypomineralization (MIH) raises the likelihood of enamel degradation, which in turn raises the risk of plaque buildup and dental caries. Individuals impacted by this illness frequently incur large long-term costs. Therefore, it is of interest to evaluate prevalence and treatment need of MIH in school going children. Hence, 3030 school going students were included in this study. Considering the WHO 1997 guidelines for caries severity and the requirement of therapy for the damaged teeth and criteria for MIH, a full mouth visual assessment of moist teeth was conducted for every student. The overall prevalence of MIH was 174 (7.9%). Preventive caries restricting therapy was needed in 42(6.2%) maxillary right first molar,30(4.5%) maxillary left first molar, 30 (4.5%) mandibular right first molar, 36 (5.4%) in mandibular left first molar. Data shows that an incidence rate of 7.4 percent was noted, with a larger propensity among male children and a predominant impact on mandibular molars.

## Background:

Disturbances in the tooth growth process throughout the maturation phase cause hypomineralization of the molars and occasionally the permanent incisors, resulting in a disorder called molar incisor hypomineralization (MIH).The impacted teeth's clinical look might range from white to brown, with a clear distinction between the enamel's normal state and its damaged areas [1, 2-3]. When biting down, the damaged enamel may be porous and readily chip off. Because of the disintegration of the enamel, the affected tooth may resemble hypoplastic; however, in situations of MIH, the uneven edges of the lesion help distinguish the tooth from hypoplasia [4, 5-6]. Because there is little force applied to the incisors, the impacted incisors simply exhibit discoloration of varied degrees and little to no enamel loss. The presence of MIH raises the likelihood of enamel degradation, which in turn raises the risk of plaque build-up and dental caries. Individuals impacted by this illness frequently incur large long-term costs [5-7]. At the community level, MIH coincides with traditional risk factors for pediatric caries and indirectly imposes a significant financial cost [8, 9-10]. The severity of the condition determines the MIH therapy options. Quick detection at the very beginning of disease along with prompt screening of children who are susceptible for MIH can result in more conservative and successful management. Globally, the overall incidence of MIH ranges from 2.4 percent to forty-two percent [11, 12, 13-14]. Therefore, it is of interest to evaluate prevalence and treatment need of MIH in school going children.

## Methods and Materials:

The study included 3030 school going students who had signed consent papers with knowledge and were in attendance on the actual date of the assessment. The dental examinations were carried out by two skilled, calibrated experts utilizing a mouth mirror and artificial light. Before starting the survey, investigators were calibrated to prevent variance in the findings. The benchmark for inter- and intra-examiner agreement was Cohen's Kappa >0.8. Considering the WHO 1997 guidelines for caries severity and the requirement of therapy for the damaged teeth, as well as the European Academy of Pediatric Dentistry (EAPD) 2003 criteria for MIH, a full mouth visual assessment of moist teeth was conducted for every student involved in the study.

## Statistical analysis:

The data was placed on MS excel sheet and subject ted for statistical analysis. The data evaluation was conducted using the Statistical Package for Social Sciences (SPSS) 21. Chi square test and ANOVA was used for statistical analysis. The distribution of the clinical observations and prevalence was subjected to a descriptive analysis. P value ≤ 0.05 was taken as statistically significant.

## Results:

The overall prevalence of MIH was 174 (7.9%). As per age distribution, MIH was observed among 34 (1.2%) in 8 year old children, 15 (1.1%) in 9 year old children, 22 (1.55%) in 10 year old children, 19 (1.35%) in 11 year old children and 14 (1.02%) in 12 year old patients. As per gender distribution MIH was observed in 100 (3.48%) boys and 74 (3.53%) girls (Table 1). No treatment was required in 68 (11.7%) maxillary right first molar, 96 (14.18%) maxillary left first molar, 74 (11.21%) mandibular right first molar, 72 (10.52%) in mandibular left first molar. Preventive caries restricting therapy was needed in 42(6.2%) maxillary right first molar,30(4.5%) maxillary left first molar,30(4.5%) mandibular right first molar, 36 (5.4%) in mandibular left first molar. It was discovered that the most prevalent phenotypic manifestation of MIH observed in children with MIH was involvement of only molars, followed by involvement of only incisors. The main treatment needed for the FPM, according to the study's findings, was the administration of pit and fissure sealants after receiving preventive as well as caries inhibiting care. The first molars' mean DMFT among the kids with MIH was identified to be 1.03. When comparing MIH-affected molars with healthy molars, it was discovered that the former had a greater clinical appearance of carious lesions (Table 2).

## Discussion:

The purpose of this research was to assess the prevalence of MH in school-age children as well as their need for treatment. The current research indicates that the total incidence of MIH in the population was 7.9%. It's probable that the exclusion of lesions with dimensions smaller than 2 mm is the cause of the research's lower occurrence. 9.2 percent frequency was identified in one research, while 7.9 percent prevalence was found in another research [10, 15- 16]. Research conducted in other places that was comparable showed a frequency ranging from 7.9 percent to 9.7 percent. According to studies conducted in many Asian countries, the prevalence was determined to be 2.8 percent, 17.6%, and 18.6% [11, 17-18]. Variations in the methods and diagnostic criteria used to evaluate the lesion may be the cause of the fluctuation in the incidence of MIH [12-19]. Data shows that boys had a higher chance of developing MIH than girls did. Gender differences in the meal choices and masticatory abilities may account for the higher frequency in males. Comparable research has shown that boys are more likely than girls to experience MIH [16, 20, 21, 22, 23-24]. Contrary to the results of the current inquiry, a Tamil Nadu research discovered that girls had a higher incidence of MIH (8.9%) than boys (6.1%) [14- 20]. The 10-year-old age group in the research investigation had a higher incidence of the MIH than other age groups. However, 13-year age range has a higher frequency of MIH [15- 24]. The greater prevalence in the 10-year-old and older age group may be explained by the ease of diagnosis, as post-eruptive coloring and breakdown are more common in older age groups than in younger ones [19- 25]. It was found that involvement of only molars alone, followed by involvement of only incisors alone, was the most common phenotypic manifestation of MIH seen in children with MIH as shown elsewhere [15- 21]. Nonetheless, a small number of research studies have suggested that involvement of both incisors and molars is more common [22-25]. This analysis revealed that mandibular right FPM was most frequently implicated, which is consistent with findings from studies on different demographic categories. Studies from China and Australia have found that maxillary molars are more commonly afflicted than mandibular molars, which runs counter to the present observation [16-24]. Data shows that maxillary incisors were more commonly affected than mandibular incisors, which is consistent with findings from other studies conducted around the globe. Dental enamel is a special tissue due to certain characteristics. It is the body's toughest tissue and contains a significant amount of inorganic materials, namely hydroxyapatite. Because of the ameloblast's poor ability to heal, disruptions that arise during the mineralization of enamel will leave behind permanent scars [11- 19]. The first molars' mean DMFT among the kids with MIH was identified to be 1.14. When comparing MIH-affected molars with healthy molars, it was discovered that the former had a greater clinical appearance of carious lesions. This is consistent with findings from a few research conducted in various parts of the globe [14- 23].

We evaluated the damaged molars' requirement for treatment based on WHO guidelines [12- 19]. It was found that most of the molars just needed to be cleaned by the patient themselves and did not require any kind of therapy. The main treatment needed for the FPM, according to the research's findings, was the administration of pit and fissure sealants after receiving preventive as well as caries inhibiting care. Applying a fifth generation bonding solution before applying sealant has been shown in an investigation to increase the rate of retention [17- 24]. Restoration on one tooth surface was more than restorations on two surfaces among the FPM requiring restorations. A few studies have suggested that for teeth afflicted by MIH, a restoration with composite resin with a conservative design and Glass Ionomer Cement (GIC) can be undertaken [15-23]. The results of this investigation showed that there was low requirement of treatment involving pulpal therapy and extraction. MIH increases the possibility of enamel deterioration, which in turn increases the risk of dental cavities and plaque accumulation. People who are affected by this condition often have significant long-term expenses [3-7]. MIH is associated with conventional risk factors for pediatric caries and carries a large financial burden on the community. The MIH therapy options are determined on the severity of the ailment [5-8]. Early diagnosis at the earliest stage of the illness and timely screening of kids at risk for MIH can lead to more successful and conservative treatment. The incidence of MIH varies between 2.4 and 42 percent worldwide. There aren't many academic researches that back MIH [7-11].

## Conclusion:

Data shows that an incidence rate of MIH was 7.4 percent was noted, with a larger propensity among male children with a predominant impact on mandibular molars. A large sample sizes is required for future research to determine the prevalence of MIH.

## Figures and Tables

**Table 1:** Prevalence of MIH according to age and gender in children

	**8 year**	**9 year**	**10 year**	**11 year**	**12 year**
MIH present	34 (1.2%)	15 (1.1%)	22 (1.55%)	19 (1.35%)	14 (1.02%)
MIH absent	500 (17.41%)	303 (20.01%)	285 (18.79%)	276 (19.10%)	324 (22.35%)
	**Boys**	**Girls**			
MIH present	100 (3.48%)	74 (3.53%)			
MIH absent	1500 (50.29%)	1376 (46.21%)			

**Table 2:** Treatment need for MIH affected molars n children

	**None**	**Preventive, caries arresting care**	**Fissure sealant**	**One surface filling**	**Two or more surface filling**	**Pulp care**	**Extraction**
Maxillary right	68 (11.7%)	42(6.2%)	28 (4.2%)	12 (1.2%)	4 (0.6%)	2 (0.2%)	4
Maxillary left	96 (14.18%)	30(4.5%)	22 (3.3%)	20 (0.9%)	10 (0.3%)	4(0.3%)	4 (0.2%)
Mandibular right	74 (11.21%)	30 (4.5%)	24 (3.6%)	24(1.2%)	14 (0.6%)	4(0.3%)	5 (0.3%)
Mandibular left	72 (10.52%)	36 (5.4%)	16 (5.3%)	28 (1.3%)	18 (1.9%)	6 (0.4%)	1
